# P-400. Overdiagnosis and Treatment of Multi-Drug Resistant Urinary Pathogens across a Pediatric Hospital System

**DOI:** 10.1093/ofid/ofaf695.617

**Published:** 2026-01-11

**Authors:** Aarika Young, Elizabeth Tocco, Tjin Koy, Grant Stimes, Denver Niles, Judith R Campbell, Lucila Marquez, Catherine Foster

**Affiliations:** Texas Children's Hospital, Houston, Texas; Texas Children's Hospital, Houston, Texas; Texas Children's Hospital, Houston, Texas; Texas Children's Hospital, Houston, Texas; Baylor College of Medicine, Houston, Texas; Baylor College of Medicine, Houston, Texas; Baylor College of Medicine, Houston, Texas; Baylor College of Medicine, Houston, Texas

## Abstract

**Background:**

Increasing antimicrobial resistance in pediatric urinary tract infections (UTI) is an area of concern and overdiagnosis of these infections may lead to unnecessary antibiotic exposure. We describe the characteristics and treatment of multi-drug resistant Gram-negative (MDRGN) UTIs in our pediatric population.

**Methods:**

We performed a retrospective review of pediatric patients seen at a pediatric hospital system in 2022 who had a urine culture positive for a MDRGN, which was defined as a Gram negative organisms that are non-susceptible to at least 1 antibiotic in ≥ 3 antibiotic groups. Organisms were considered extended spectrum β-lactamase (ESBL) producers if their resistance profile showed resistant to ceftriaxone or ceftazidime. We determined the proportion of patients treated for a UTI who were overdiagnosed according to AAP clinical practice guidelines. Clinical and microbiologic data was obtained from the electronic medical record. Descriptive statistics and Fisher’s exact were used for analysis.

**Results:**

One hundred and forty-one children had 190 urine cultures positive for a MDRGN. *Escherichia coli* (128, 67%) and *Klebsiella* spp. (44, 23%) were isolated most frequently followed by *Serratia* (7, 4%)*, Enterobacter* (5, 3%)*, Citrobacter* spp. (3, 2%) and one each of *Proteus mirabilis, Acinetobacter baumannii,* and *Morganella morganii*. Patients with MDR E. coli compared to all other MDRGNs, were significantly less likely to have neurogenic bowel and bladder (*P*< 0.001) while there was no observed difference in vesicoureteral reflux (*P*=0.3) or history of UTI antibiotic prophylaxis (*P*=0.4). Most patients with a urinalysis had evidence of pyuria (136/171, 80%). Of 190 urine cultures, 159 (84%) had a single organism and 136 (72%) had colony count of > 50,000 CFU/mL. Most of the *E. coli* (111, 87%) and *Klebsiella* (42, 95%) isolates were ESBL producers. Of 169 episodes treated as a UTI, 79 (47%) did not meet guideline criteria. Conversely, two patients meeting criteria were not treated.

**Conclusion:**

Escherichia coli was the predominate MDRGN found in urine cultures. Almost half of the patients treated for an infection did not meet UTI definition according to national guidelines, highlighting an opportunity for antibiotic stewardship.

**Disclosures:**

Denver Niles, MD, BioMerieux: Advisor/Consultant

